# Supplementary material for: Long-Read RNA Sequencing Identifies Polyadenylation Elongation and Differential Transcript Usage of Host Transcripts During SARS-CoV-2 In Vitro Infection
Source: Front Immunol. 2022 Apr 6;13:832223. doi: 10.3389/fimmu.2022.832223 (PMC9019466; doi:10.3389/fimmu.2022.832223)
Supplement: Supplementary Table 1 — poly(A) and poly(T) datasets (r < 0.4), with all correlations being significant (p-value < 0.05, Pearson’s correlation test). These results indicate that median poly(A) and poly(T) lengths from direct cDNA preparations can differ per gene and that one of the two datasets may be a better predictor for true poly(A) lengths. Related to Figure S3 and Table 4 . . [file Table_1.docx]

| Cell line | Time point | r | P-value |
| --- | --- | --- | --- |
| Caco-2 | 2 | 0.247 | < 2.2 x 10^-16^ |
| Caco-2 | 24 | 0.208 | 1.873 x 10^-7^ |
| Caco-2 | 48 | 0.302 | < 2.2 x 10^-16^ |
| Calu-3 | 2 | 0.233 | < 2.2 x 10^-16^ |
| Calu-3 | 24 | 0.213 | 1.785 x 10^-13^ |
| Calu-3 | 48 | 0.267 | <2.2 x 10^-16^ |
| Vero | 2 | 0.370 | < 2.2 x 10^-16^ |
| Vero | 24 | 0.249 | 2.043 x 10^-11^ |
| Vero | 48 | 0.279 | 2.524 x 10^-14^ |
