# Supplementary material for: Long-Read RNA Sequencing Identifies Polyadenylation Elongation and Differential Transcript Usage of Host Transcripts During SARS-CoV-2 In Vitro Infection
Source: Front Immunol. 2022 Apr 6;13:832223. doi: 10.3389/fimmu.2022.832223 (PMC9019466; doi:10.3389/fimmu.2022.832223)
Supplement: Supplementary Table 2 — Comparison of significantly differentially polyadenylated non-mitochondrial gene clusters in the Calu-3 48 hpi datasets between nanopolish, tailfindr poly(A) and poly(T) outputs (before log-transformation) using Wilcoxon’s test of ranks. nanopolish and tailfindr poly(T) results were more comparable compared with tailfindr poly(A) results, as no significant polyadenylation was observed in tailfindr poly(A) data. These results suggest that the tailfindr poly(T) lengths may be more suitable for estimating differential polyadenylation compared with tailfindr poly(A) lengths. Related to Figures S3 and S4 and Table 4 . [file Table_2.docx]

| Direction of Polyadenylation | *nanopolish* (nonMT) | *tailfindr* poly(A) (nonMT) | *tailfindr* poly(T) (nonMT) |
| --- | --- | --- | --- |
| Up | 457 | 0 | 72 |
| Down | 3 | 0 | 1 |
| Total | 460 | 0 | 73 |
