# Supplementary material for: Long-Read RNA Sequencing Identifies Polyadenylation Elongation and Differential Transcript Usage of Host Transcripts During SARS-CoV-2 In Vitro Infection
Source: Front Immunol. 2022 Apr 6;13:832223. doi: 10.3389/fimmu.2022.832223 (PMC9019466; doi:10.3389/fimmu.2022.832223)
Supplement: Supplementary file 7 [file Presentation_1.pdf]

Supplementary Material

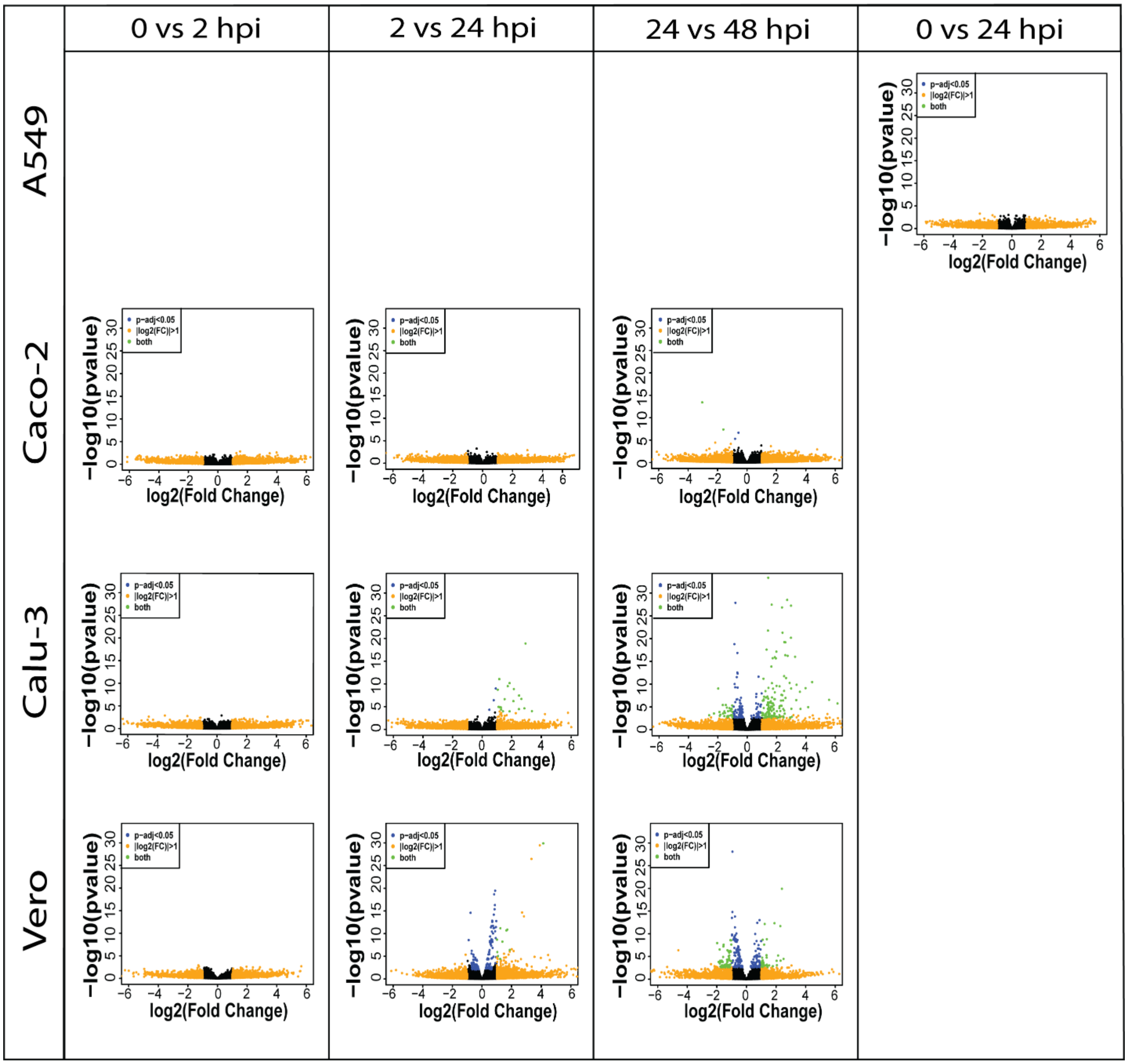

Figure S1. Volcano plots showing changes in differential expression between control and infected cells across two time points in direct cDNA datasets using *DESeq2* and the interactive term. X-axis represents log2FC and Y-axis displays –log10 p-value, • padj < 0.05, • |log2(FC)| > 1, • both. Plots reveal increase in differential expression at later time points compared with earlier time points in Caco-2, Calu-3 and Vero cell lines, and a lack of significant differential expression was observed between A549 0 vs 24 hpi. These results resemble the data generated in **Figure 1**, where A549 does not present with changes in gene expression level, and Vero and Calu-3 cells present more changes in gene expression level compared with Caco-2 at later time points. These results show the cell-type-dependent responses to SARS-CoV-2. Related to **Figures 1, 2** and **Table 2**.

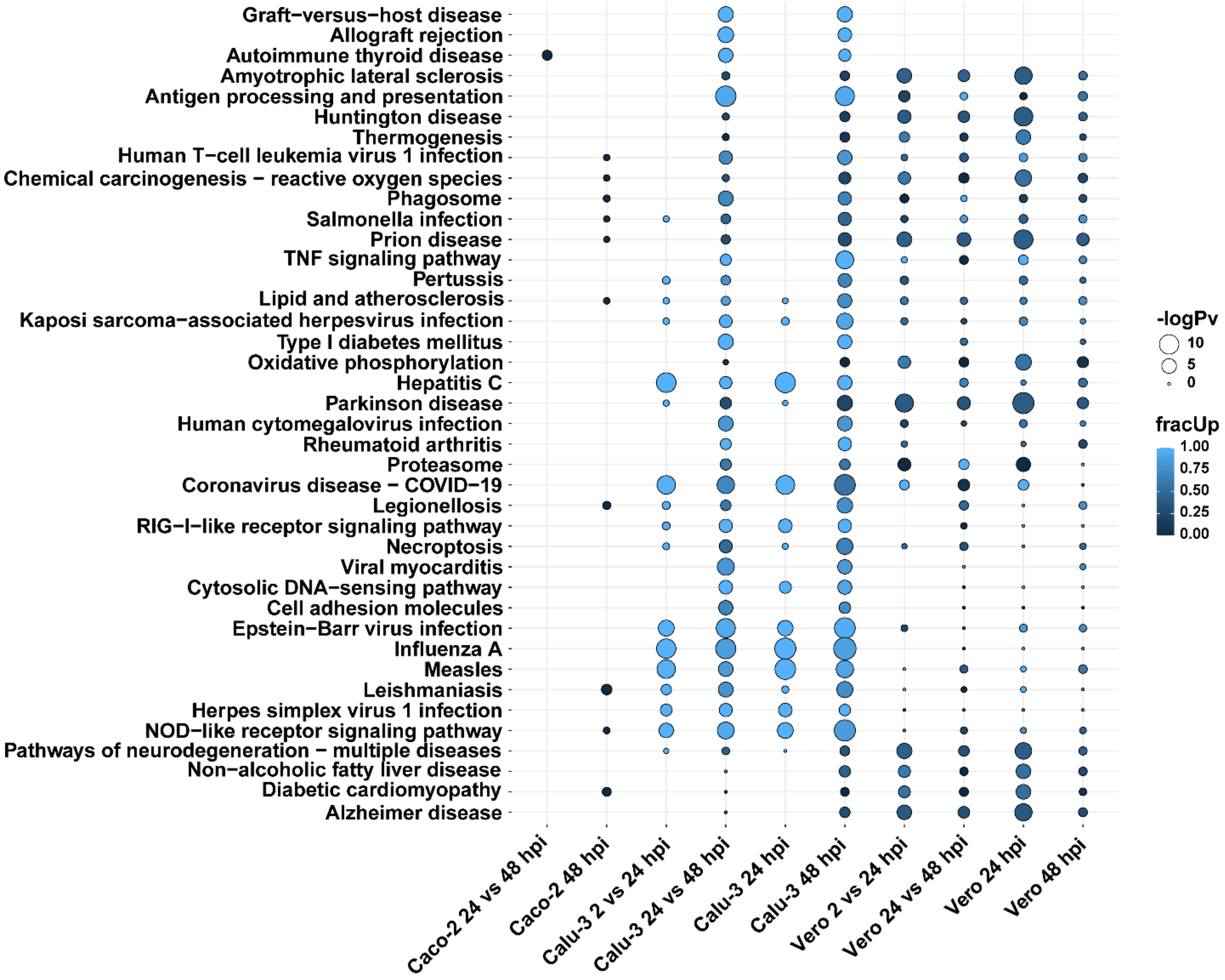

Figure S2. KEGG pathways from differentially expressed genes in Calu-3, Caco-2 and Vero direct cDNA datasets analyzed by *DESeq2*. Results include datasets (in order); Caco-2 24 vs 48, Caco-2 48, Calu-3 2 vs 24, Calu-3 24 vs 48, Calu-3 24, Calu-3 48, Vero 2 vs 24, Vero 24 vs 48, Vero 24 and Vero 48 hpi. Strongest KEGG pathway enrichment was observed in the Calu-3 cells including pathways such as *influenza A*, *measles* and *coronavirus diseas*e. These results reveal that Calu-3 cells show strong anti-viral responses which are typical for viral infections such as SARS-CoV-2 infections compared with Caco-2 and Vero cells. The bubble size and color indicate the -log10 enrichment p-values and fraction of upregulated genes, respectively. Thresholds of padj < 0.05 and enrichment p-value < 0.0001 in at least one dataset were used for generating the plot, and all terms with padj < 0.05 and enrichment p-value < 0.05 were deemed as significant for the analysis. Related to **Figure 3, Table 3** and **Data S2**.

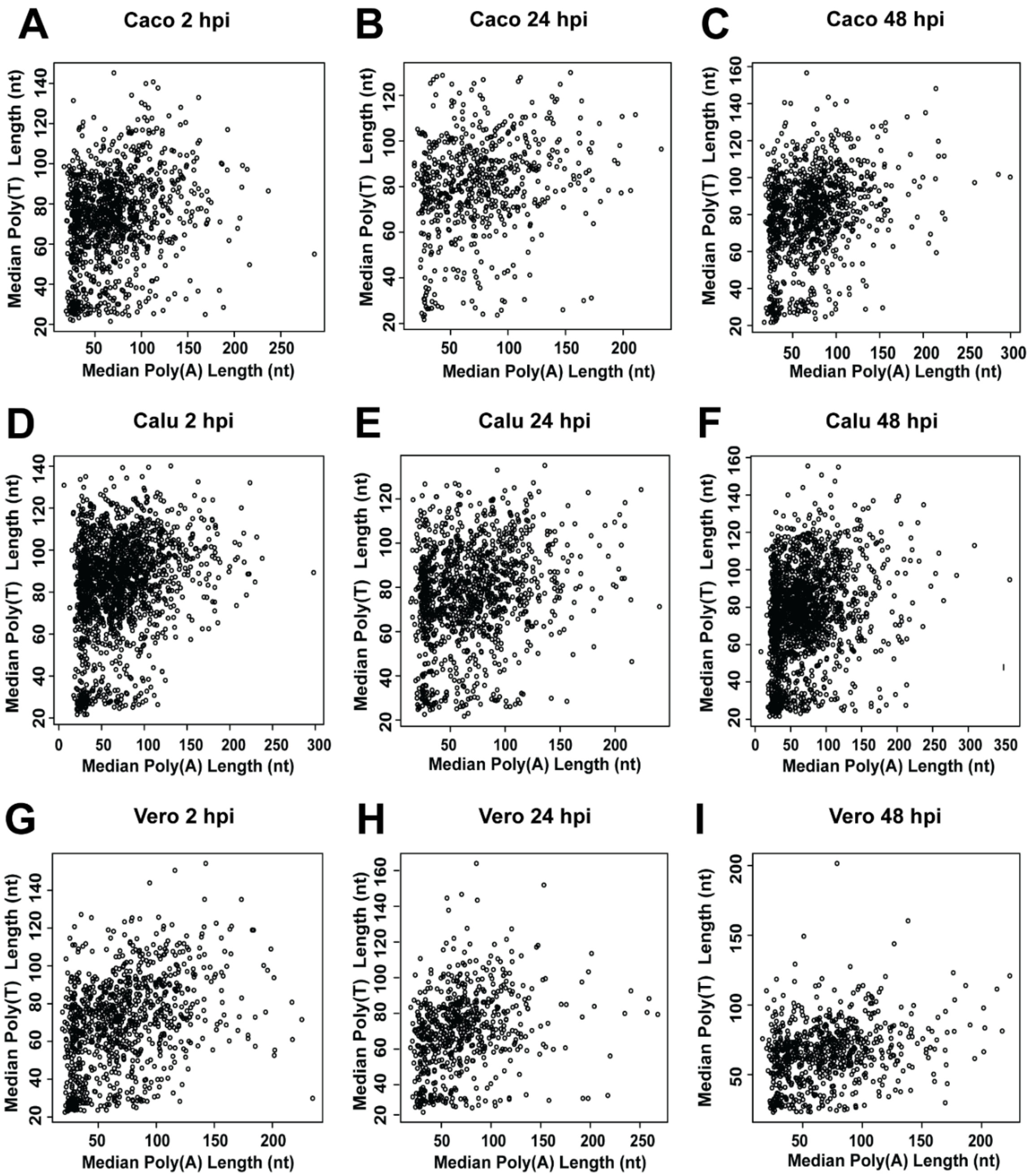

Figure S3. Correlations between median poly(A/T) lengths from *tailfindr* analyses. Scatter plots comparing *tailfindr* poly(A) vs poly(T) datasets from Caco-2 **(A)** 2 hpi, **(B)** 24 hpi, **(C)** 48 hpi, Calu-3 **(D)** 2 hpi, **(E)** 24 hpi, **(F)** 48 hpi and Vero **(G)** 2 hpi, **(H)** 24 hpi**, (I)** 48 hpi. Weak correlations between median poly(A) and poly(T) lengths were observed ($R < 0.4$, exact values along with p-values are indicated in **Table S1**), where each dot represents a gene. These results indicate that median poly(A) and poly(T) lengths from direct cDNA preparations can differ per gene and that one of the two datasets may be a better predictor for true poly(A) lengths. Related to **Tables 4, S1, S2**.

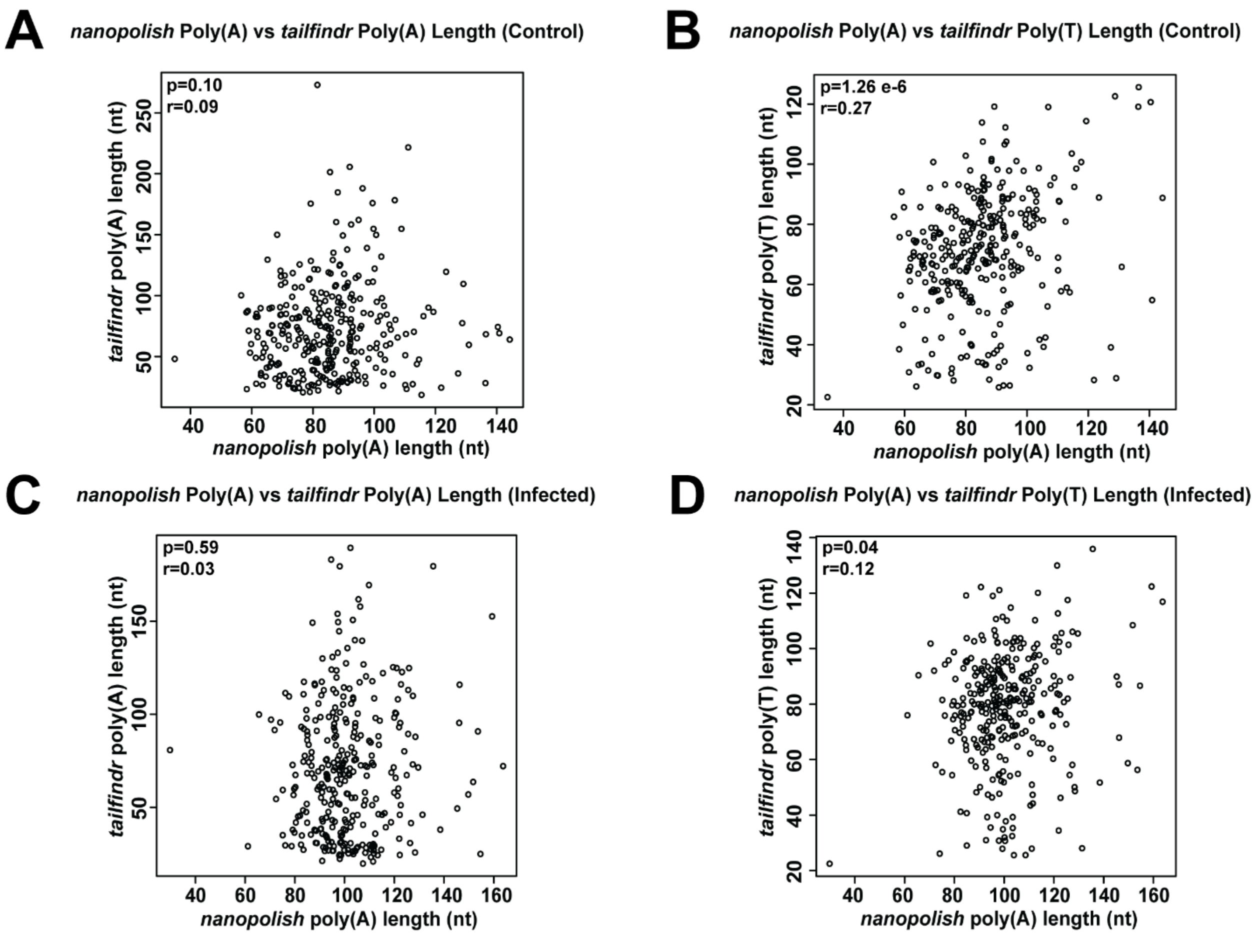

Figure S4. Correlations between median poly(A) lengths from *nanopolish* and poly(A/T) lengths from *tailfindr* analyses. Scatter plots comparing **(A)** median *nanopolish* poly(A) and *tailfindr* poly(A) lengths in control cells **(B)** median *nanopolish* poly(A) and *tailfindr* poly(T) lengths in control cells, **(C)** median *nanopolish* poly(A) and *tailfindr* poly(A) lengths in infected cells and **(D)** median *nanopolish* poly(A) and *tailfindr* poly(T) lengths in infected cells. Weak significant positive correlations were observed between the *nanopolish* poly(A) and *tailfindr* poly(T) datasets (r = 0.12-0.27, p-value < 0.05, Spearman's correlation test). No significant correlation was observed between *nanopolish* poly(A) and *tailfindr* poly(A) datasets (p-value > 0.05, Spearman's correlation test). These results reveal that *tailfindr* poly(T) lengths from direct cDNA datasets are better estimators of *nanopolish* poly(A) length from direct RNA datasets compared with *tailfindr* poly(A) lengths. Related to **Tables 4, S2**.

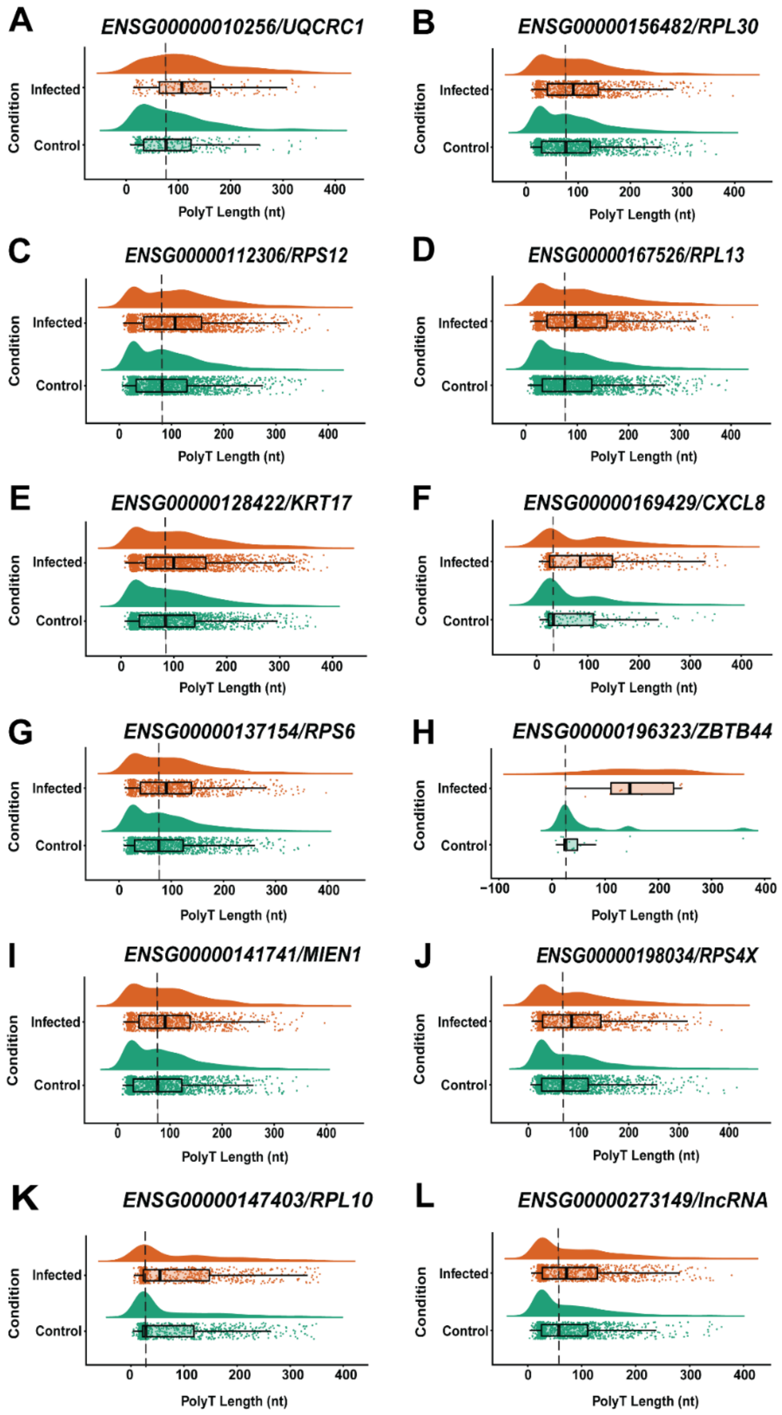

Figure S5. Raincloud plots of raw, untransformed poly(T) tail lengths in twelve genes with increased poly(A) tail length in the *tailfindr* poly(T) mixed-model analysis with log-transformation from the Calu-3 48 hpi dataset. Genes include **(A)** *UQCRC1*, **(B)** *RPL30*, **(C)** *RPS12*, **(D)** *RPL13*, **(E)** *KRT17*, **(F)** *CXCL8*, **(G)** *RPS6*, **(H)** *ZBTB44*, **(I)** *MIEN1*, **(J)** *RPS4X*, **(K)** *RPL10* and **(L)** a lncRNA. These plots suggest that SARS-CoV-2-infected Calu-3 cells have increased mRNA poly(A) lengths compared with control cells at 48 hpi. Related to **Figure 4**.

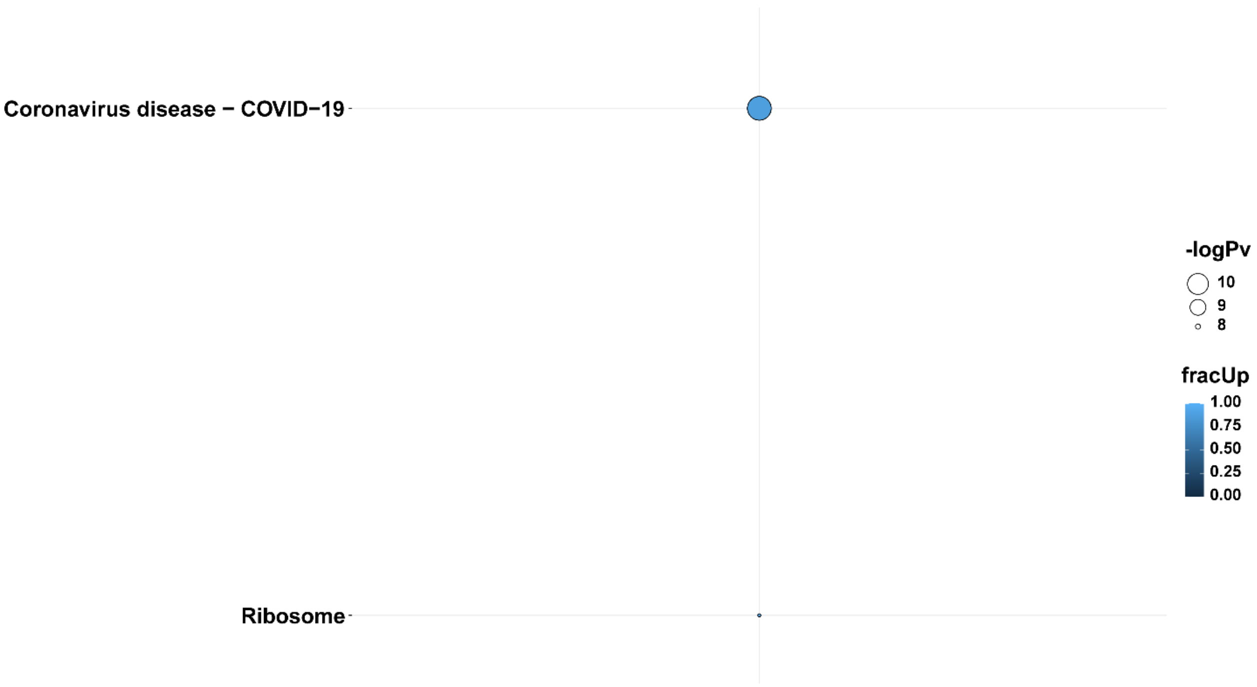

Figure S6. KEGG pathways from genes with differential poly(A) tail length in *tailfindr* poly(T) mixed-model analyses from the Calu-3 48 hpi dataset. Genes involved in *coronavirus disease* and *ribosome* pathways were increased in poly(A) length after infection. These results indicate that genes with differential polyadenylation are involved in the coronavirus disease and ribosome pathways, which suggests a potential role for polyadenylation in host defense or viral manipulation of host processes during SARS-CoV-2 infections. The bubble size and color indicate the -log10 enrichment p-values and the fraction of genes with increased polyadenylation, respectively. Thresholds of padj < 0.05, enrichment p-value < 0.0001 were used. Only bubbles which meet the thresholds are shown. Related to **Figure 4** and **Data S3**.

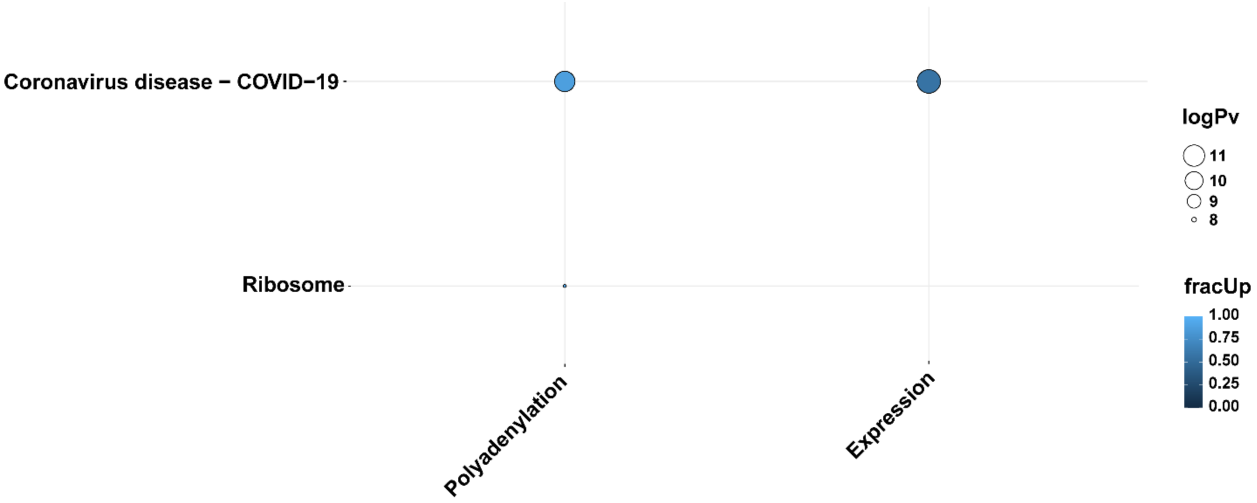

Figure S7. KEGG pathways of differentially polyadenylated and expressed genes from the Calu-3 48 hpi direct cDNA dataset. Only the *coronavirus disease* pathway was shared between the two analyses, as enrichment of the ribosome pathway was not significant in the differential expression analyses. The plot shows a potential correlation in increased poly(A) tail length and downregulation in gene expression. This phenomenon may arise due to the virus-driven translation inhibition and host-driven post-transcriptional regulation. The bubble size and color indicate the -log10 enrichment p-values and fraction of upregulated genes/genes with increased polyadenylation, respectively. Thresholds of padj < 0.05, enrichment p-value < 0.0001 were used. Related to **Figure 5** and **Data S4**.
